# Towards the Three Dimensions of Sustainability for International Research Team Collaboration: Learnings from the Sustainable and Healthy Food Systems Research Programme

**DOI:** 10.3390/su132212427

**Published:** 2021-11-10

**Authors:** Rashieda Davids, Pauline Scheelbeek, Nafiisa Sobratee, Rosemary Green, Barbara Häesler, Tafadzwanashe Mabhaudhi, Suparna Chatterjee, Nikhil Srinivasapura Venkateshmurthy, Georgina Mace, Alan Dangour, Rob Slotow

**Affiliations:** 1 Centre for Transformative Agricultural and Food Systems, School of Agriculture, Earth and Environmental Sciences, University of KwaZulu-Natal, Pietermaritzburg 3201, KwaZulu-Natal, South Africa; 2 Centre on Climate Change and Planetary Health, London School of Hygiene & Tropical Medicine, London WC1E 7HT, UK; 3 Department of Pathobiology and Population Sciences, Royal Veterinary College, London E16 2PX, UK; 4 Ashoka Trust for Research in Ecology and the Environment Royal Enclave, Sriramapura, Jakkur Post, Bangalore 560064, India; 5 Public Health Foundation of India, Gurgaon 122002, India; 6 India and Centre for Chronic Disease Control, New Delhi 110016, India; 7 Department of Genetics, Evolution and Environment, University College London, London WC1E 6BT, UK; 8 School of Life Sciences, University of KwaZulu-Natal, Pietermaritzburg 3201, KwaZulu-Natal, South Africa

**Keywords:** sustainability, climate change, carbon footprint, virtual conference, transdisciplinary team, virtual team, learning

## Abstract

This paper highlights the potential for learning and virtual collaboration in international research teams to contribute towards sustainability goals. Previous research confirmed the environmental benefits of carbon savings from international virtual conferences. This paper adds the social and economic dimensions by using a combination of qualitative and quantitative methods to measure the constraints and benefits for personal development, economic costs, efficiency and team learning of holding international virtual conferences (VCs). Using the Sustainable and Healthy Food Systems (SHEFS) research programme as a case study, we analysed VC participant survey data to identify strengths, weaknesses, opportunities, and threats of VCs. We estimated ‘saved’ GHG emissions, costs, and time, of using VCs as an alternative for a planned in-person meeting in Chennai, India. Hosting VCs reduced North–South, gender, and researcher inclusivity concerns, financial and travelling time costs, and substantially reduced emissions. For one international meeting with 107 participants, changing to a virtual format reduced the per capita GHG emissions to half the annual global average, and avoided 60% of travel costs. The benefits of VCs outweighed weaknesses. The main strengths were inclusivity and access, with 20% more early/mid-career researchers attending. This study identified opportunities for international research partnerships to mitigate their carbon footprint (environmental benefit) and enhance inclusivity of early/mid-career, women and Global South participants (social benefit), whilst continuing to deliver effective collaborative research meetings (economic benefit). In doing so, we present a holistic view of sustainability opportunities for virtual collaboration.

## 1 Introduction

As research scientists, we are tasked with paving the way towards a more sustainable future. The United Nations has for decades worked with countries and the scientific community to develop policies and plans aiming to protect people and the planet, and achieve sustainability through environmental protection, social development and economic growth [1–4]. Each Strategic Plan, up to and including the 2020 Agenda for Sustainable Development and the sustainable development goals (SDGs), recognised that, to achieve sustainable development, socio-economic outcomes for basic human well-being such as poverty alleviation and the reduction of inequalities, are essential. However, equally important is the achievement of environmental goals, including urgent actions against climate change [3]. Evidence-based research is geared to identify prospects to enhance opportunities, and mitigate risks, to achieving the sustainable development goals (SDGs) [3], and targets for limiting global warming to within 1.5 °C above pre-industrial levels [5]. Despite frequently having first-hand knowledge of sustainability and climate change challenges, the international research community still contributes significantly towards greenhouse gas emissions. For example, American ecologists were found to have carbon footprints over twice that of average Americans, and more than ten times the global average (4.5 tonnes of CO_2_ equivalents (eq) a year) in 2009, predominantly due to greenhouse gas emissions from air travel for one international collaborative meeting [6]. A single astronomers’ conference in Europe held in 2019 was roughly equal to India’s average annual per capita emissions [7]. More recently, enforced mandatory confinement during the COVID-19 pandemic majorly restricted business and leisure travel, resulting in a huge decline of CO_2_ emissions in the first half of 2020 compared to 2019 (−17%) [8]. However, the drop in carbon emissions was short-lived, with the overall decline for 2020 being only 6% [9].

Scientists have become weary of the problem and responsibility of mitigating emissions associated with hosting international conferences [7]. Previous studies have confirmed the reduction of carbon emissions as a major benefit of hosting virtual vs. face-to-face international academic conferences in light of COVID-19 travel restrictions [7,10,11]. However, reducing carbon emissions is only one part of the three dimensions needed for scientists to contribute towards sustainability: a complete solution must focus on social, economic and environmental sustainability, as called for in the Sustainable Development Agenda [12].

A ‘virtual team’ involves interactions amongst a group of people, from different offices, places or times zones, working together on interdependent tasks towards a common goal, whereas ‘virtual collaboration’ is the process by which virtual teams complete collective tasks and achieve common goals [13]. Virtual project teams have become more common due to globalisation and project-based work of many contemporary organisations [14], with an explosion of virtual team research over the past decade [15]. The broad research focus has included understanding the effectiveness of virtual teams over face-to-face teams [16], overcoming issues of trust, identity [17] and knowledge sharing [18] and adapting to transition from collocated to online COVID-19 working conditions [19]. Although some research has focussed on the environmental sustainability contributions of virtual team collaboration, mainly from a carbon savings perspective [10,11,20], this study is the first to holistically also consider the other dimensions of sustainability for virtual collaboration.

Transformational changes are needed across all sectors to ensure that we bridge the science-action gap [21], actively mitigate climate change [22], and achieve global sustainability. The application of sustainability frameworks to better understand the linkages between anthropogenic actions, environmental limits and social outcomes [22–24], can be used to ensure that all three dimensions of sustainable development are considered. With adequate planning, international research teams can contribute to multiple sustainable development goals (SDGs), for example; SDG 5: Achieve gender equality and empower all women and girls; SDG 10: Reduced inequalities within and among countries; SDG 13: Take urgent climate action to combat climate change and its impacts; SDG 16: Promote peaceful and inclusive societies for sustainable development and build effective and inclusive institutions; and SDG 17: Strengthen global partnership for sustainable development. The international research community could take the lead by learning and acting upon the evidence that is generated around finding solutions, and inspire others to follow their example [6].

Despite virtual teams gaining increasing popularity, there remains uncertainty regarding the effectiveness of virtual teams over face-to-face teams [16]. Whilst there are some obvious benefits in terms of avoided costs, greenhouse gas emissions, and travel time, other more hidden advantages could contribute to sustainability. Virtual meetings could, for example, contribute to the social domain through increasing inclusivity, mainly related to attendance by researchers from the Global South, particularly females, and by early-career researchers, for whom travel budget could restrict their face-to-face participation more often than for their counterparts from the Global North. Academic literature describes the gender bias, where women have been found to publish and participate in collaborations less than their male counterparts, particularly in science, technology engineering, mathematics and medicine [25]. Furthermore, scientists with care duties could be restricted in time spent away from home but would be able to participate virtually. This calls for solutions to barriers causing inequality, often faced by women and early-career researchers in science [26].

Although there has been an upsurge in virtual collaboration due to the availability of collaborative technology, the abandonment of face-to-face interaction has its challenges [13]. The six major challenges identified from over a decade of virtual collaboration research are the virtual team ‘givens’ of technology, time and distance, and the virtual team ‘creations,’ of trust, culture and leadership [13]. Drawbacks of virtual meetings include lack of personal contact, restricted possibilities for networking, and total reliance on IT equipment. In order to ensure high-performance or sustainability of virtual teams, the challenges of both these ‘givens’ and ‘creations’ need to be addressed [13].

This research explored the various benefits and constraints of virtual communication and collaboration, with the view to identify barriers and opportunities for virtual teams to contribute to sustainability. This study investigated the three domains of sustainability frameworks plus an additional learning domain, by asking the following key questions: (1) Social: How can learning and collaboration in virtual teams assist in enhancing inclusivity for marginalised scientists, such as those in the Global South, or early-career researchers who may be constrained by funding; or women? (2) Environmental: How can international research teams effectively use virtual collaboration to actively contribute to global sustainability and climate change mitigation efforts? (3) Economic: What are the benefits and costs of hosting international research meetings, and how can they be enhanced or reduced? (4) Learning: How can systems thinking principles assist in unpacking learnings and improving virtual research collaboration processes going forward? To answer these, we used a mixed methodological approach, combining quantitative and qualitative data: learning organisation surveys and Strengths, Weaknesses Opportunities and Threats (SWOT) analyses, greenhouse gas emissions and cost analyses to compare holding large-scale multi-site and multi-disciplinary virtual conferences (VCs), over face-to-face in-country meetings. We analysed international conferences and research team dynamics of the Sustainable and Healthy Food Systems (SHEFS) research programme as our case study.

## 2 Materials and Methods

### 2.1 Description of Case Study

SHEFS is a multi-disciplinary boundary organisation operating across three countries: South Africa, the United Kingdom, and India. SHEFS aims to influence policy towards achieving sustainable food systems that deliver improved health outcomes and reduced environmental impacts [27]. The SHEFS research programme includes 13 institutions, with over 100 academics, government practitioners, and other stakeholders, from over 20 different disciplines within and related to the agriculture-environment-health nexus. Over four years of research, SHEFS has produced more than 80 internationally published research papers contributing to the food system, environment, and health domains [28]. This study formed part of a PhD undertaken by the lead Author, in her capacity as a SHEFS researcher and programme manager.

#### Background to SHEFS International Meetings

SHEFS started in 2017, and annual meetings have been hosted since, with staff and students from each country site personally attending the first two meetings held in London, United Kingdom (2017), and Durban, South Africa (2018). In the face of an increasing awareness of the climate costs of meeting physically, the SHEFS management team decided to host the Annual Meeting in 2019 via a VC, in place of the originally planned inperson meeting in Chennai, India. This meeting offered a unique opportunity to determine whether operations within the programme could be conducted more sustainably in terms of social development, costs, time, and carbon footprints, whilst maintaining or improving upon the level of group learning and engagement previously experienced in face-to-face conferences.

For the first VC in 2019, the team assembled physically in groups in five virtual rooms (one in the UK, two in India, two in South Africa), plus several individuals were joining from their personal computers. Zoom (Barbosa et al., 2019) virtual meeting software was used for communication during the conference, with some of the preparatory work recorded using Microsoft Collaborate [29]. Conference organisers identified innovative ways to increase opportunities for engagement at the VC. First, several presentations were recorded ahead of the meeting. Participants were encouraged to watch pre-recorded presentations and send questions and comments to the presenter ahead of the meeting. The “live” time during the VC was then used for more in-depth discussion.

Furthermore, presenters were encouraged to make use of interactive tools, such as Mentimeter [30], to facilitate active participation during the conference. In each of the “physical” rooms, a venue-leader was assigned, who registered any potential contributions (questions, comments, etc.) of participants in their respective rooms, and alerted the moderator of a session accordingly. Hand raising and ‘question and answer’ typing functions of the Zoom software were used in addition to this.

In March 2020, a second virtual meeting was held, with all participants attending virtually and individually, as the COVID-19 pandemic restricted movement and face-to-face meetings. The VC linked 73 participants from South Africa, United Kingdom, and India. Learnings from the first VC allowed for more effective preparation, and, this time, SHEFS early-career researchers from each country planned and prepared the agenda and conference activities before the VC. Multiple activities were facilitated for engagement and direct discussions of research before the conference, namely ‘journal club discussions’ which allowed participants to meet virtually to discuss publications (SHEFS outputs); ‘feedback workshops’ with in-depth discussions for problem-solving and enhancement of specific research projects; and ‘presenter of another team member’s output’ where participants discussed the research of another researcher, to present the outputs to the broader team during the VC. Online presentations were delivered via the Zoom platform during the conference. Breakaway ‘meeting rooms’ (in Zoom), linked to the VC, were used for small group discussions. Up to five participants were able to brainstorm particular topics before returning to the main virtual room for plenary feedback.

Involvement in the above meetings provided the authors and research team with the necessary background and experience to assess the benefits and constraints of VCs, analysed in this study.

### 2.2 Methods

**Figure 1:**
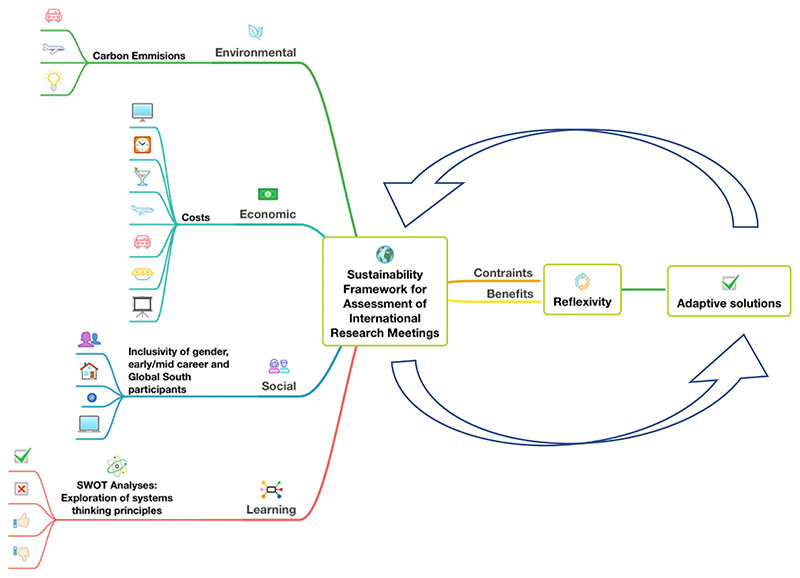
Methodologies used to compare virtual vs. face-to-face meetings. Environmental: Estimation of greenhouse gas emissions in kg CO_2_ equivalents, from flights, using ClimateCare 2012. Economic: Calculation of costs for flights, venue hire, food, beverages, airport transfers and lodging. Social: Participant online surveys, analysis of participants (career level, gender, North vs. South) and survey data. Learning: Analyses of strengths, weaknesses, opportunities, and threats (SWOT), to identify measures to enhance project outcomes and mitigate threats of hosting VCs. Quantitative environmental and economic data combined with qualitative social and learning data, to develop a holistic view of benefits vs. constraints of VC, allowing for reflexivity and identification of adaptive solutions to challenges and opportunities for improving sustainability.

This study used mixed methods to analyse broad-ranging aspects of hosting virtual vs. face-to-face international research meetings, related to the three broad themes of sustainability plus a learning dimension, namely, (1) Environmental: analysis of greenhouse gas emissions, (2) Social: inclusivity of gender, early-career and Global South participants, (3) Economic: costs of hosting virtual vs. face-to-face meeting; and (4) Team learnings and reflexivity (Figure 1). We used and analysed both qualitative (social and team learning: surveys) and quantitative (environmental and economic: GHG and cost) data, with the view to understand the benefits and challenges of virtual meetings by reflecting on the VC system as a whole [31].

Our social analysis (surveys) focussed on the 2019 and 2020 virtual meetings (*n* = 4), with surveys undertaken before and after each meeting (*n* = 4) (Appendix A). The greenhouse gas calculations and costs for the environmental and economic components considered only the 2019 VC, as it had the highest number of attendees for all the meetings.

#### 2.2.1 Environmental: Greenhouse Gas Emissions

We estimated the transport-related GHG emissions for the face-to-face conference that would have occurred if the 2019 virtual conference had been held in Chennai as initially planned. This was done to estimate the reductions in carbon footprint achieved by holding the 2019 conference virtually. We used the preferred flying route of the researchers—often a combination of flight time and costs—to calculate the distance from their respective locations to Chennai. Assuming economy class flights, we used the ClimateCare carbon calculator [32] to estimate flight emissions in kg CO_2_ equivalents. The methods used by the ClimateCare calculator have been published elsewhere [32], but in short: the calculator estimates the orthodromic distance between two airports and estimates associated carbon emissions. Additional multipliers are applied for first or business class, long-haul flights (>3700 km), and for flying at high altitudes (the radiative forcing index multiplier). We excluded the consideration of internet-related emissions, as these were found to be negligible in other studies, calculated at less than 0.1% of total emissions for a face-to-face VC [7]. Similarly, per person daily network and laptop average emissions for attending a VC in a similar study accounted only for 0.03% of total emissions compared to face-to-face [7].

#### 2.2.2 Economic: Costs

We estimated the flight prices at economy class fares (prices listed in August 2019) for each researcher who indicated that they were attending the annual meeting in Chennai in person, and compared these against costs incurred for the 2018 annual meeting, held in South Africa. Additionally, we included venue hire, food and beverages, airport transfers, and lodging costs of all attendees in the “Chennai scenario.” We did not consider local hotel-to-venue commuting costs, nor the “usual” home-work costs for the virtual scenario. We included costs for equipment hire, needed for the online meeting for each institution—if not yet in place.

#### 2.2.3 Social: Inclusivity of Gender, Youth and Global South Participants

We listed the level of seniority, including early-career (postgraduate students), mid-career (defined as researchers below Associate Professor level or equivalent) and senior level of each attendee of the virtual meeting, and proposed attendees of the Chennai meeting (should the meeting have been held face-to-face), and compared the proportion of early- and mid-career level attendees between the two scenarios. We also calculated the percentages of attendees, and their genders, from the Global South, which consisted of team members from South Africa and India, for both scenarios.

#### 2.2.4 Learning: Participant Surveys and SWOT Analyses

To analyse the perceptions of participants before and after the virtual conferences, two online surveys were conducted using SurveyMonkey for each meeting. The surveys comprised both multiple choice and open questions, and aimed at capturing participants’ perceptions of the advantages and disadvantages of the VCs.

The authors identified and discussed the emerging main and sub-themes, and data were extracted and categorised/coded by theme. Four authors reviewed the data and reached a consensus on coding to ensure intercoder reliability [33]. Themes were summarised using participants’ quotes as an illustration, and Strength (S), Weaknesses (W), Opportunities (O), and Threats (T) (SWOT) were identified in each main and sub-theme. Each SWOT was ranked based on its significance, calculated using an online SWOT analysis tool (Mind Doodle, 2018).

The significance (or scores) of Strengths (S) and Weaknesses (W) were calculated as a product of ‘importance,’ ranked on a scale of 1 (Low/minimal effect)—5 (High, vitally important) and ‘internal rating,’ ranked on a scale of 1 (Minor, could be done better/do not do it too poorly)—3 (Major, excel at this/do it poorly).

The significance of the Opportunities (O) and Threats (T) was calculated as a product of ‘importance,’ and ‘likelihood,’ ranked on a scale of 1 (Low, unlikely)—3 (Major, highly likely). The results were displayed in a bubble graph to show the relative significance of each SWOT (Appendix B, Figure A1). Weaknesses and threats were assigned negative scores for display purposes on the graph (Appendix B, Figure A2).

We explored the learning component by assessing systems thinking principles [31] of our learning organisation through qualitative causal loop analysis. This was done to further understand the impact of the large-scale, multi-site, and multi-disciplinary virtual processes, to explore the underlying forces at play when considering research collaboration, and to express the potential for systems thinking to facilitate finding adaptive solutions, in response to identified challenges in virtual collaboration. Interlinkages among the SWOT analysis components, reflexivity [34], and ongoing learning were heuristically expressed to demonstrate how learning can lead to desired change, i.e., mitigating identified weaknesses and threats to successful collaboration and partnership, thereby enhancing project outcomes.

## 3 Results

**Figure 2:**
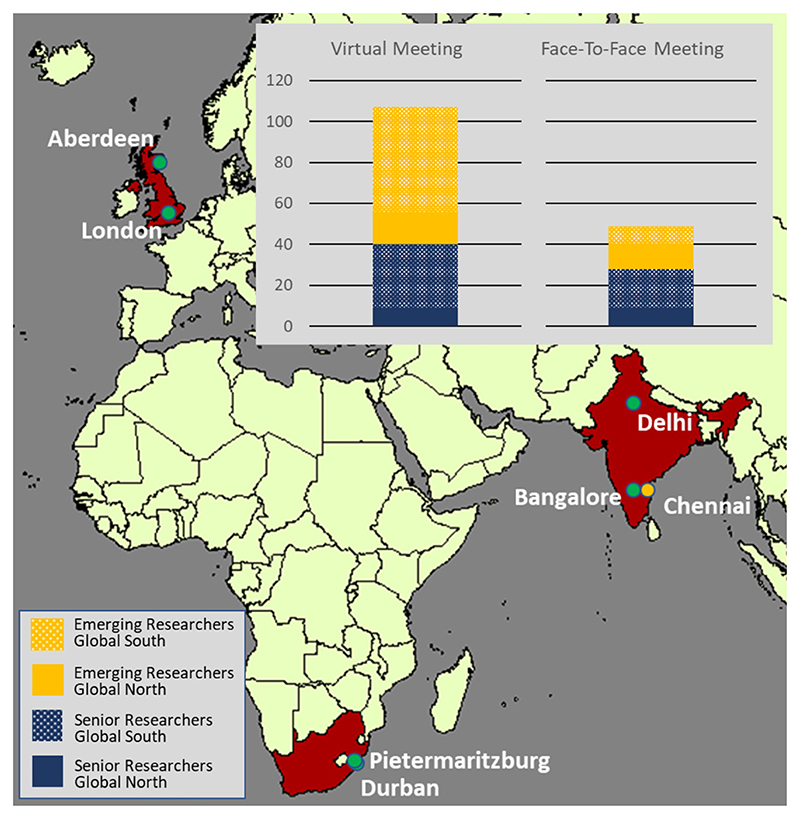
Location of research institutions (green), numbers and level of seniority of researchers that attended the 2019 VC and that would have attended the face-to-face meeting in Chennai (orange).

In total, 107 researchers attended the virtual meeting in October 2019. Of these, 49 indicated that they would also have attended a physical meeting if this would have taken place in Chennai (Figure 2). The numbers by location can be found in Appendix C (Table A1). In total, 63 participants completed the survey before the start of the meeting and 41 the “after” 2019 survey.

### 3.1 Environmental Footprints and Costs

Carbon footprints of 37 international flights and 12 national flights, plus airport transfers, were estimated to be a total 123,009 kg CO_2eq_, should the meeting have been held face-to-face with 49 participants. This would have amounted to 2.5 tonnes of CO_2eq_ per attendee, which is just over half of the global annual average footprint of a single person in 2009 (Fox et al., 2009). The total flight time of all researchers combined was estimated to have been 881 h, and the total travel time 1080 h (i.e., 45 person-days); thus, an average of 22 h per person was saved by holding the meeting in a virtual format.

**Figure 3:**
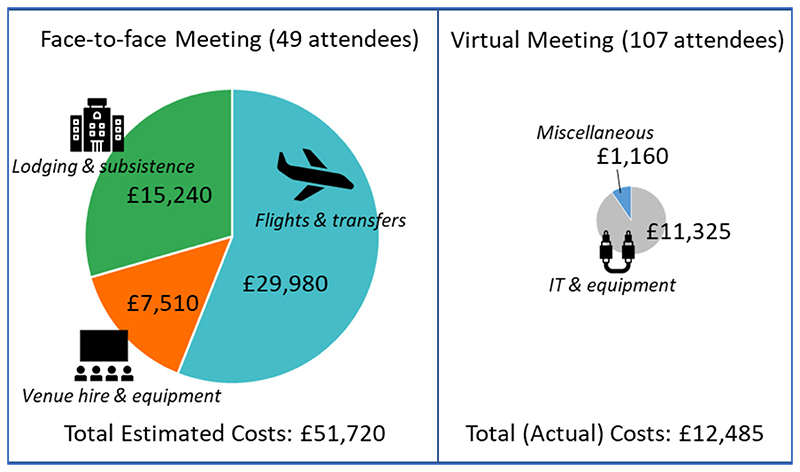
Estimated conference costs for face-to-face left and virtual meeting (right) for 2019 VC. Should the VC have been held face-to-face in Chennai, only 49 team members would have attended due to budgetary constraints.

Total costs associated with the 2019 face-to-face meeting were estimated to be GBP 51,720. Approximately 60% of these costs involved air travel (Figure 3). Actual costs related to virtual annual meeting attendance were GBP 12,485 for all institutions combined, of which the majority (GBP 11,325) was spent on equipment hire and purchase. Furthermore, the amount used in purchasing equipment during this initial virtual meeting was a one-time investment, and the equipment purchased could be used for subsequent VCs, unlike costs incurred in air travel, which would keep rising in the subsequent in-person meetings. Incidentally, there were no equipment costs for the VC in South Africa, as this was already available at the institution. The average per-person cost of GBP 1055 for the initially planned face-to-face meeting with 49 attendees decreased to GBP 117 per person in the virtual meeting in which 107 researchers participated.

Costs, greenhouse gas emissions and travel time commitments related to the preceding 2018 face-to-face meeting in Kloof, South Africa (57 participants) were slightly lower on average per person than the estimated figures of the Chennai meeting due to more in-country participants, totalling GBP 26,573 (GBP 466 per person) (Exchange rate R16.97/GBP 1 at the time of the meeting), 92,475 kg CO_2eq_ (1.6 tonnes of CO_2eq_ per person) and 880 h (15.4 h per person). For the South Africa meeting, the flights accounted for 83% of the costs.

In 2017 and 2018 the SHEFS research community held annual meetings; however, the shift to a virtual mode allowed conducting bi-annual meetings. This allowed for more frequent interaction and allowed researchers from across countries to share their work and get feedback in a more efficient manner.

### 3.2 Inclusion and Participation: Gender and Global South

For the 2018 meeting, a total of 57 people attended, of which 30 were from South Africa (including 5 external South African policy stakeholders external to the SHEFS team, who attended part of the meeting), 25 from the UK and 2 from India. Of attendees, 22 (39%) were early-career researchers (9 from Global South and 13 from Global North) whilst 35 (61%) were senior (23 from Global South and 12 from Global North).

**Figure 4:**
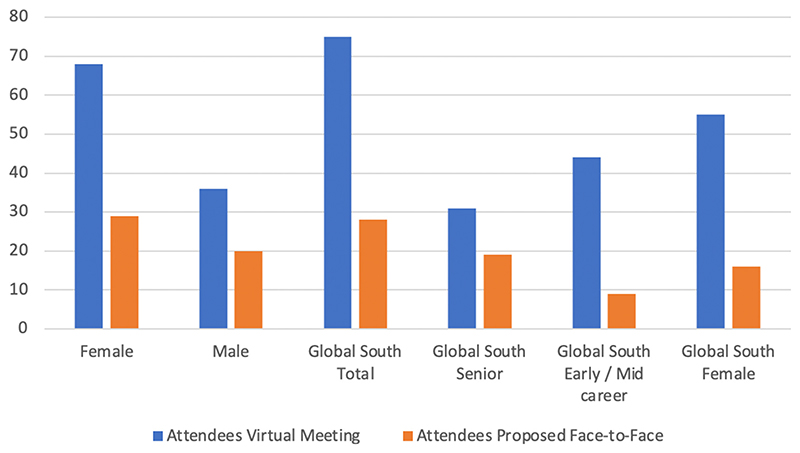
Gender and Global South attendees comparisons for virtual vs. proposed face-to-face conference in 2019.

In 2019, of the 107 participants that attended the virtual meeting, 63% (67) were early-career researchers, of which 59% (44) were from the Global South. In the case of the face-to-face meeting, this would have been 43% (21), with 42% (9) from the Global South (Figure 4, Appendix C). In terms of gender, the number of female participants that attended was 65% (68), compared to 59% (29) that would have participated at the face-to-face meeting. Of these, 78% (53) females who attended were from the Global South, compared to 55% (16) that would have attended the face-to-face meeting.

### 3.3 Learnings and SWOT Analyses

**Figure 5:**
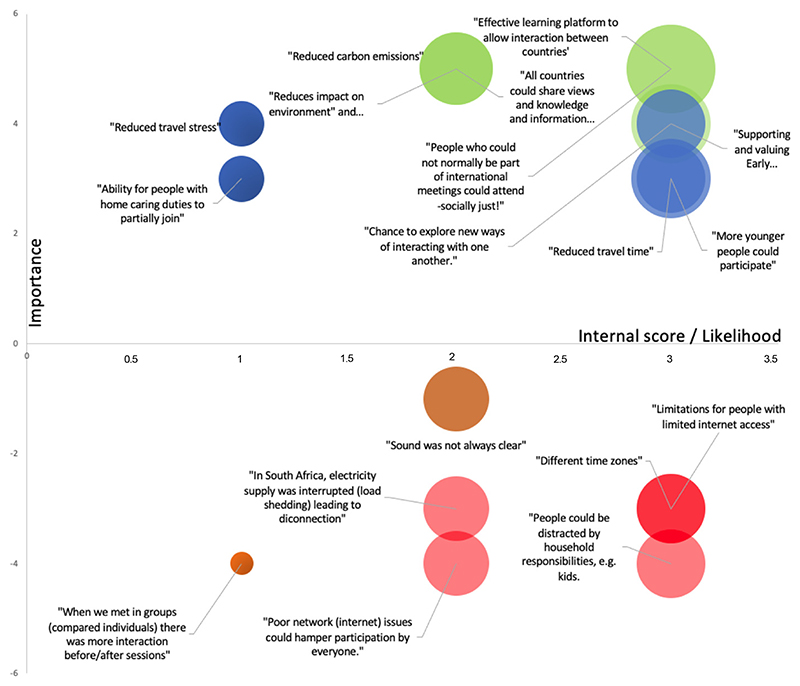
SWOT Analyses: Plotted based on the value of importance (X-axis), internal rating (for Strengths (green) and Weaknesses (brown) or likelihood for Opportunities (blue) and Threats (red)) (Y-axis). The size of the bubbles signify the significance (total scores) of SWOT. Only the top seven SWOT were shown here.

We identified SWOT from open-ended comments participants made on their perceptions of the virtual meetings; 14 strengths, three weaknesses, 12 opportunities, and nine threats, and assigned scores for each (Appendices B and D). We identified three main themes from these: (1) project productivity, (2) personal development, and (3) opportunities for participation. Within these, we identified ten sub-themes (Appendix D, Figure A3). Figure 5 shows the top seven Strengths, Weaknesses, Opportunities, and Threats, in relation to each other.

#### 3.3.1 Strengths (S)

The most significant strengths were under the ‘participation’ theme. Enhanced opportunities to participate and increased inclusivity was a recurrent comment in the surveys, especially by early-career scholars from the Global South. Furthermore, despite the limitations of the virtual meeting format, social interaction was frequently mentioned as a strength, particularly for communication across countries. This included positive views of this type of virtual communication for research progress.

“[…] people who normally could not be part of international meetings could attend—socially just approach !!!”—Senior researcher, Global South

“The virtual meeting format is an effective learning platform that allows interaction between countries.”—Senior researcher, Global South

“It is convenient and easy. All countries can share their views, knowledge and information in one “room” thus saving travelling costs”—Early-Career Researcher, Global South

“[…] we could engage and share with each other in very challenging times, students of mine logged in to the conference from some of the most remote places in South Africa and just loved being part of the learning experience […]”—Senior Researcher, Global South

Other key strengths of hosting the VC were under ‘personal development’ related to personal time management and active contribution to a low carbon economy. With the research consortium focusing on sustainability issues, the reduced environmental footprint of the VC was a frequently mentioned sub-theme, and seen as a major strength of the VC format. Participants indicated that they appreciated the fact that, in this way, they were themselves “actively” contributing to lowering environmental footprints.

“Significantly lower carbon footprint for the meeting and, thus, for the SHEFS project as a whole.”—Senior Researcher, Global North

“In the current time frame, where the effects of climate change are becoming frequent and more calamitous, virtual conferences are one of the ways to reduce our carbon footprint.”—Mid-Level Researcher, Global North

“It was less disruptive to my workday to be able to join individually”—Early-Career Researcher, Global North

“The benefit was [that] this was logistically useful as it saved a lot of valuable time which would otherwise be spent in travelling and upsetting schedules. This initiative was also feasible at a carbon-footprint level”—Early-Career Researcher, Global South

#### 3.3.2 Weaknesses (W)

In terms of weaknesses, under the ‘participation,’ theme it was felt that social interaction was hindered at the 2020 VC, where everyone met via Zoom as individuals during COVID-19, compared to the 2019 VC (where countries met virtually in groups). Other weaknesses related to technical issues, such as a weak internet connection, were mentioned numerous times, and identified as threats in SWOTs.

“…When we met in groups (compared to individuals) there were more interactions before/after sessions, but the experience was pretty similar for me during the actual sessions.”—Senior researcher, Global North

“[Disadvantages of hosting the VC was] Not being able to have the direct connection and social interaction. Not being able to ask how people are really doing. Not being able to ask more sensitive questions to someone after a nice meal when the mood is relaxed and people have built some rapport. All the small human connections as social beings that make use of all non-verbal cues.” — Senior researcher, Global North

“I wish more time could have been given to some of the discussions as they were very interesting”—Senior Researcher, Global South

#### 3.3.3 Opportunities (O)

Far more opportunities were identified than threats. Many opportunities were highlighted related to ‘work-life balance,’ for ‘personal well-being,’ most significantly that attending VCs resulted in reduced travel stress, the ability for more early-career researchers and people with home caring duties to participate, and a saving of personal time and energy.

“If the meeting had been held in person I wouldn’t have been able to go (as I have a young child), but with a virtual meeting I am able to attend.”—Senior Researcher, Global North

“More younger people could participate…More engagement by participants. Empowering for different sites as they could all participate and influence”— Senior Researcher, Global South

“[…] Better use of time, resources (money and natural) and energy (human)… Allows part-time workers to engage etc. Just so many wins.”—Senior Researcher, Global North

Opportunities related to ‘social interaction’ were also noted, where participants felt that the VCs provided a platform to explore new ways of connecting with each other on equal terms. Other comments were centred around the ability of the VC to facilitate continued ‘research progress’ despite the COVID-19 pandemic, and that the VC enabled ‘progressiveness and innovation’ related to learning and use of new technical skills and tools.

“It will give a chance to connect members from different places and they can share their opinions and have discussions live. Annual meeting can be left online and be accessible in future”—Early-Career Researcher, Global North

“Maintaining a sense of community and partnership despite [the COVID-19] pandemic. Keeping partnerships strong and driving forward research. Supporting and valuing Early-Career Researchers.”—Senior Researcher, Global North

“[…] Scientific side of the meeting was as good/better than face-to-face. Great for widening participation and access.”—Senior Researcher, Global North

#### 3.3.4 Threats (T)

Threats were identified under each main theme, most of which fell under the ‘logistical efficiency’ and ‘time productivity’ sub-themes. The fact that the VC had to consider different time zones across South Africa, the United Kingdom, and India meant that the conference duration for each day needed to be limited to four hours. This was about half of the time allocated for the face-to-face conference. This threat was compounded in the 2020 VC by ‘time productivity’, whereby participants mentioned that household distractions hindered their participation. Other issues raised were related to ‘social interaction,’ limited time for personal interactions, and poor internet connectivity.

“People could be distracted by household responsibilities, for example, kids”—Early-Career Researcher, Global South

“More difficult to remain focussed when everything is online”—Early-Career researcher, Global North

“I think the limited time also meant that new partnerships did not have enough time to be formed”—Senior Researcher, Global South

“Sometimes the sound was not very good. It was harder to have real back-and-forth discussions”—Early-Career researcher, Global North

“The internet connectivity in my area was terrible and this meant that I missed parts of the meeting”—Early-Career researcher, Global South

### 3.4 Heuristic Model of SWOT Analyses

**Figure 6:**
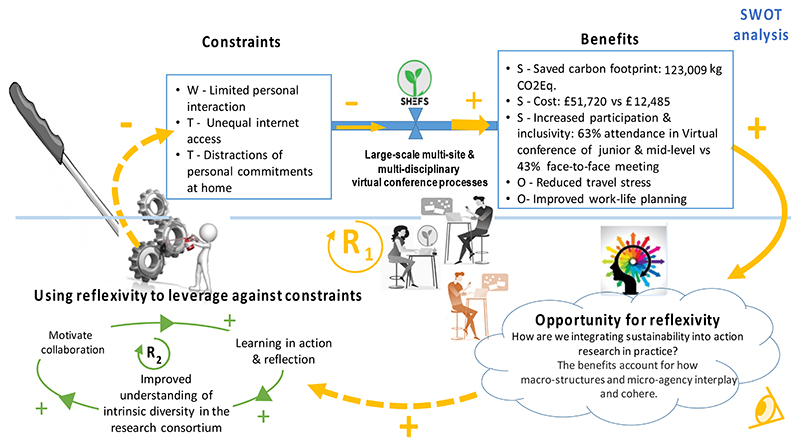
A heuristic model of multi-disciplinary virtual conferences as a constituent form of sustainability practice. The output of the SWOT analysis is shown in the upper half of the diagram, whilst when considered together with the lower second half, the systemic perspective, as a virtuous reinforcing loop (R_1_), is designated. Solid yellow arrows: what was found through the surveys. Blue linkage highlights that virtual meeting processes give rise to benefits and constraints. Yellow dashed arrows: how to harness the virtual meeting processes to leverage against constraints. Green arrows: Core of learning processes required for effective collaboration, denoted through a virtuous reinforcing sub-loop, R_2_. S = Strengths, W = Weaknesses, O = Opportunities, T = Threats.

The main reinforcing loop (R1 in Figure 6) highlights the interconnections among the meetings as a set of processes enabling reflexive thinking through the interplay of the linkages between various aspects of the collaborative system, namely, the benefits, constraints, opportunities for reflexivity and responses to learnings. These linkages, through learning, can be leveraged to enhance benefits and address constraints associated with the virtual meetings. Reflexivity, here, relates to how the virtual meeting processes, including the surveys, enable the researchers to evaluate how, whilst trying to achieve a specific set of sustainability objectives through the lens of sustainability, they are, in turn, actively contributing towards other aspirational goals, such as reduction of the carbon footprint through reduced international travel and social development of women and early-career researchers. The heuristic model shows that the process of learning is iterative, and only through learning and reflecting, and then amending actions, can processes of collaboration be improved.

## 4 Discussion

Debates around the effectiveness of virtual vs. face-to-face meetings have either shown virtual meetings as lacking effectiveness (particularly for building trust), or suitably able to meet the objectives of face-to-face meetings [16]. Our study contributes to evidence that with proper planning, understanding the benefits and constraints related to contextual organisational dynamics, and finding adaptive solutions, virtual collaboration can serve as a good alternative to face-to-face meetings [35]. This study shows three major sustainability advantages of virtual meetings above face-to-face meetings: significant contribution to avoiding greenhouse gas emissions (environmental); enhanced participation by early-career scholars and women, especially from the Global South, and lower barriers for inter-country interaction, communication, and collaboration (social) and significant reduction in costs (economic). By considering the whole system, we expressed the holistic view of the pros and cons of virtual collaboration, and the possibilities for virtual teams to be reflexive, and therefore able to address challenges and enhance opportunities through finding adaptive solutions.

Positive environmental effects such as reduced carbon are not expected to continue in the post-COVID-19 era, however, the pandemic showed the great potential for improved environmental health from redesigning multi-institutional collaboration and communication for reduced travel [36]. Since the SHEFS research community already conducted the VC in October 2019 and was planning for the one held in March 2020, many challenges of suddenly shifting to virtual meetings, because of COVID-19 lockdown restrictions, were avoided.

The option to join virtually allowed more than double the number of females and early-career researchers from the Global South to attend than would have attended the face-to-face conference. Our findings show that virtual collaboration can assist in bridging research science gaps, such as the North-South divide (for example, 10:1 ratio of scientific and technical articles produced in 2011 were by Northern vs. Southern authors) [37] and the gender gap (for example, 87 of 115 article disciplines examined had fewer than 45% women authors) [25]. Specifically, the VC opened up opportunities for inclusion and equal participation of more early-career researchers, Global South scientists, and women. In so doing, virtual collaboration can be used as an additional tool to address gender biases in science [26].

Estimated cost savings of hosting VCs were substantial, with an approximate 76% reduction, the majority of which was from flights. Other costs, not assessed in this study, include lengthy and financially burdensome visa applications to attend international conferences, most of which are hosted in the Global North and are thus unaffordable for many Global South researchers [38]. Utilising part of the foregone travel costs to build better infrastructure in places where it is lacking could ensure further inclusivity and participation improvement.

Effective research planning is crucial for research progress through VCs, and a number of trade-offs, such as limited possibilities to network, lack of opportunities for personal interaction, technical difficulties, and distractions/disengaging from the meeting, were experienced by participants. Some trade-offs will likely be resolved or tackled over the next few years: with faster connectivity (such as fibre internet and 5G networks) being rolled out across the world, mentioned IT and connectivity problems could become less of a problem in the near future. However, other trade-offs, and particularly those related to social interaction and face-to-face networking, which have been found to be crucial for developing trust and bonding social capital in business [39], are more complicated to overcome.

Certain threats appear to have more impact on early-career to mid-career researchers, compared to senior researchers, which may be intrinsically linked with the nature of the weaknesses and threats mentioned by the researchers. This was due to limited finances or fewer previous opportunities to build relationships or network. Senior researchers typically have had more face-to-face meetings in the past years (or decades) to build up their networks, whilst early-career researchers are yet to establish their collaborations. The option of hosting participants in regional groups, in each location, can address the threat of limited opportunities to network whilst achieving avoided air travel [35].

By effectively planning opportunities around VCs for personal interaction between participants, VCs present several strengths and opportunities that not only enhance research efficiency and potential but also provide opportunities for enhancement of personal well-being of researchers [15]. Our study also supports the use of hybrid communication options: part of the reason for the success of the VCs presented here can be attributed to the hybrid nature of SHEFS, having had foundational personal face-to-face interactions and learnings before engagement in VCs, which allowed for interpersonal relationships to be built. The ongoing fostering of such relationships, including aspects of trust and shared understanding, is critical, and we show that virtual communication can effectively be used for this purpose [13]. Another contributor to the success was that the participants were in locations where time differences between countries are not too great (India, SA and UK). The VC model may not work if the locations are too far away (for example, US and India 9.5 h to 12.5 h difference).

Whilst solutions to sustainable development challenges are predominantly, and rightly, based on science [3], there is a need to give equal emphasis to the learning processes whilst conducting research, to contribute new solutions in a complementary way. Iterative reflection and learning of all participants in transdisciplinary teams should be encouraged [40], to continually evolve towards active achievement of improved sustainability outcomes. By analysing participant feedback, and through sharing of possibilities as they emerge (for example, through new interactive tools), the research experience can be further enhanced, and high-quality research collaboration can be maintained whilst reducing costs and improving research sustainability.

The challenge resides in successfully demonstrating the occurrence of concepts, such as reflexivity, that strengthen virtual research collaboration by applying a constructivist perspective [41]. As such, the SHEFS programme has overarching interdisciplinary objectives and is a complex space for collaboration. The inclusion of the virtual meeting processes promote participation in concrete problem-solving, experimentation, and learning techniques, which eventually improve the researchers’ reflexivity [34]. Considering context specificity is essential when trying to sustain complex virtual meetings across sites, as it could influence the gap between short- and medium-term outcomes and perceptions of inclusivity and participation. For instance, not all organisations had the optimal technology arrangement for hosting virtual meetings.

Some limitations of the study include that the results reflect a case study for which the boundary organisation, SHEFS, is already focused on achieving sustainability outcomes. This may have influenced the responses by participants, specifically, their positivity towards hosting the VC and the identification of the ‘strength’ of the VC for reducing the environmental impact of the meeting. Other limitations include that the surveys were taken voluntarily, and, thus, the entire team was not represented. Despite these limitations, this study has relevance for planetary health research, policy, and practice. The case study does represent a “field study” which has been in operation since 2017 (as opposed to experimental studies on virtual collaboration) and thus offers a credible view on the ability of virtual teams to overcome challenges and achieve good outcomes [16]. Specifically, hosting VCs over face-to-face conferences can contribute to sustainability and the achievement of multiple SDGs, and the social, economic and environmental benefits outweigh the trade-offs. However, multiple improvements are needed, namely, investing in efficient IT equipment; planning for conferences to include more time for interpersonal connections, albeit online; including sufficient engagement activities during the meeting to mitigate threats of distractions or lack of focus by participants; facilitating enhanced networking for early-career researchers; finding the right balance of face-to-face vs. VCs, that is acceptable to research funders; and sharing of learnings through scientific publications.

## 5 Conclusions

This study confirms that virtual collaboration can contribute to environmental, economic and social sustainability, namely: (1) Virtual communication and collaboration have many benefits that—in several circumstances—appear to outweigh the constraints posed by the lack of face-to-face interaction, especially in times of severe disruptions, such as experienced in the ongoing COVID-19 pandemic; (2) Virtual collaboration is critical to reducing carbon emissions of the international scientific community; and (3) Virtual teams are more inclusive of marginalised scientists, such as those in the Global South, or early-career researchers who may be constrained by funding, or women, (4) VCs provide massive cost and time savings, which offer opportunities for use in other areas of research, and development, of researchers. This paper highlights that VCs can successfully enable continued progress of transdisciplinary research and achieve climate and sustainability goals, despite physical distances between team members. The transformative approach, based on using technology more fully, and effective planning to accentuate strengths and opportunities, and to mitigate weaknesses and threats, provided platforms for inclusion, participation, and influence on the project outputs and outcomes, vastly improving the innovation, robustness, and application of the science.

Although the current global situation in some way forces research collaboration to take place virtually [10], the benefits of VCs must not be forgotten if and when the pandemic ceases. At that time, it would be incumbent upon the research community to reflect on the multiple benefits for people and the planet, and the strengths and opportunities of VCs, that outweigh the weaknesses and threats.

## Supplementary Material

Appendix

## Data Availability

The data presented in this study are available on request from the corresponding author.
