# Supplementary material for: Towards the Three Dimensions of Sustainability for International Research Team Collaboration: Learnings from the Sustainable and Healthy Food Systems Research Programme
Source: Sustainability. Author manuscript; Available in PMC 2023 Sep 8. (PMC7615057; doi:10.3390/su132212427)
Supplement: Appendix [file EMS187384-supplement-Appendix.pdf]

Appendix A

Appendix A.1. Questionaires

Appendix A.1.1. Pre-Annual Meeting Survey Questions 2019

1. This survey will be done before and after the meeting. Please add your name (or code name) for tracking in the comment box below.
2. When you first heard that the Annual Meeting would be held via Virtual Conference, what was your reaction?
3. What do you see as benefits of hosting the Annual Meeting via Virtual Conference?
4. What do you see as challenges/disadvantages of hosting the Annual Meeting as a Virtual Conference?
5. Based on the planned Agenda, do you feel there are any aspects that could be improved upon to increase the success of the Annual Meeting?
6. What do you see as reasonable objectives to be achieved though the Annual Meeting? Please rate the level of achievement you predict, given the format of the meeting being by Virtual Conference (High, Moderate, Low, Not sure).
    a Share knowledge and information
    b Network
    c Facilitate deeper collaboration between organisations
    d Develop new links between organisations
    e Showcase SHEFS capability to have policy impact
    f Achieve SHEFS objectives and outcomes
    g Develop deeper understanding of the food-health-environment nexus
    h Bridge the science-action gap
    i Build trust and mutual understanding among partners
    j Gather new ideas for exploring similar research and/or implementation in my country
    k Other (please specify).
7. Do you have any other general comments about the Annual Meeting 2019?

Appendix A.1.2. Post Annual Meeting Survey Questions 2019

1. his survey will be done before and after the meeting. Please add your name (or code name) for tracking in the comment box below.
2. After experiencing the Virtual Conference, what was your reaction?
    a I was pleased.
    b I was disappointed.
    c It didn't matter to me.
3. What did you see as benefits of hosting the Annual Meeting via Virtual Conference?
4. What did you see as challenges/disadvantages of hosting the Annual Meeting as a Virtual Conference?
5. Based on the Agenda, do you feel there were any aspects that could be improved upon to increase the success of the Annual Meeting in future?
6. Which of the following objectives do you feel were achieved during the Annual Meeting 2019 Virtual Conference? Please rate the level of achievement you experienced (High, Moderate, Low, Not sure).
    a Share knowledge and information
    b Network
    c Facilitate deeper collaboration between organisations
    d Develop new links between organisations
    e Showcase SHEFS capability to have policy impact
    f Achieve SHEFS objectives and outcomes
    g Develop deeper understanding of the food-health-environment nexus
    h Bridge the science-action gap

- i Build trust and mutual understanding among partners
- j Gather new ideas for exploring similar research and/or implementation in my country
- k Other (please specify).

7. Do you have any other general comments about the Annual Meeting 2019?

Appendix A.1.3. Pre-Annual Meeting Survey Questions 2020

1. This survey is part of a series of surveys being done as part of SHEFS meetings. Please add your name (or code name) for tracking in the comment box below.
2. The last virtual annual meeting was held in October 2019. When you heard that SHEFS is hosting the next Virtual Meeting in March 2020, and that it would be more often (twice instead of once per year), what was your reaction?
   - a I was pleased
   - b I was disappointed
   - c It didn't matter to me.
3. Given that all three countries are in Lockdown due to COVID-19, the format of the virtual meeting has changed from "meeting rooms" to "individuals" linking in via Zoom. What do you see as reasonable objectives to be achieved though this format of the Bi-Annual Meeting? Please rate the level of achievement you predict (High, Moderate, Low, Not sure).
   - a Share knowledge and information
   - b Network
   - c Facilitate deeper collaboration between organisations
   - d Develop new links between organisations
   - e Showcase SHEFS capability to have policy impact
   - f Achieve SHEFS objectives and outcomes
   - g Develop deeper understanding of the food-health-environment nexus
   - h Bridge the science-action gap
   - i Build trust and mutual understanding among partners
   - j Gather new ideas for exploring similar research and/or implementation in my country
   - k Other (please specify).
4. What do you see as benefits of hosting the Bi-Annual Meeting via Virtual Conference, linking in as individuals due to COVID-19 Lockdown?
5. What do you see as challenges/disadvantages of hosting the Bi-Annual Meeting as a Virtual Conference, linking in as individuals during COVID-19 Lockdown?
6. Prior to the Annual Meeting, various activities were arranged to increase information sharing, collaboration, interaction and feedback between SHEFS Colleagues. Please ascribe the level of effectiveness for each activity that was conducted (High, Moderate, Low, Not sure).
7. Journal club discussions
   - a Presenter of someone else's output
   - b Research feedback workshop meetings
   - c Other (please specify)
8. Do you have any other general comments about the Bi-Annual Meeting 2020?
9. How has COVID-19 affected your work-life balance?
   - a I have less time to work
   - b I have more time to work
   - c My fear around the virus has overpowered my capabilities for usual work functions
   - d I have been able to focus more and reflect on doing things better
   - e My household responsibilities have taken preference

- f I am ill or am taking care of ill relatives
- g I'm spending more time assisting people in my community to cope with COVID-19
- h No change
- i Please add any "other" response below, or elaborate on your responses above.

10. Do you have any suggestions for SHEFS to contribute solutions to the COVID-19 crisis?

Appendix A.1.4. Post Annual Meeting Survey Questions 2020

1. After experiencing the Bi- Annual Virtual Conference during the COVID-19 Lockdown, what was your reaction?
   - a I was pleased
   - b I was disappointed
   - c It didn't matter to me.
2. Prior to the Annual Meeting, various activities were arranged to increase information sharing, collaboration, interaction and feedback between SHEFS Colleagues. Please ascribe the level of effectiveness for each activity that was conducted (High, Moderate, Low, Not sure).
   - a Journal club discussions
   - b Presenter of someone else's output
   - c Research feedback workshop meetings
   - d Other (please specify)
3. Given that all three countries are in Lockdown due to COVID-19, the format of the virtual meeting was changed from "meeting rooms" to "individuals" linking in via Zoom. What did you experience as reasonable objectives that were achieved through this format of the Bi-Annual Meeting? Please rate the level of achievement.

   Share knowledge and information

   Network
   - a Facilitate deeper collaboration between partners
   - b Develop new links between organisations
   - c Showcase SHEFS capability to have policy impact
   - d Achieve SHEFS objectives and outcomes
   - e Develop deeper understanding of the food-health-environment nexus
   - f Bridge the science-action gap
   - e Build trust and mutual understanding among partners
   - h Gather new ideas for exploring similar research and/or implementation in my country
   - i Other (please specify)
4. What did you see as benefits of hosting the Bi-Annual Meeting via Virtual Conference?
5. What did you see as challenges/disadvantages of hosting the Annual Meeting as a Virtual Conference?

   How often would you prefer SHEFS to host Virtual Conferences of this nature?
   - a Bi-Annually
   - b Quarterly
   - c Annually
6. How did your experience differ, from linking in to the Virtual Conference in groups as done in October 2019, compared to linking in as an individual this time?
7. Do you have any suggestions for SHEFS research that could offer solutions to some of the challenges presented by COVID-19?

8. Do you have any other general comments about the Bi-Annual Meeting March 2020?

Appendix B

(a) SWOT Bubble diagram and (b) SWOT Analyses Scores Table. The bubble diagram was drawn in MindDoodle, using the significance calculations for each SWOT in the Table, as calculated using the formula:

Score = Significance/Importance × Internal ratings/Likelihood

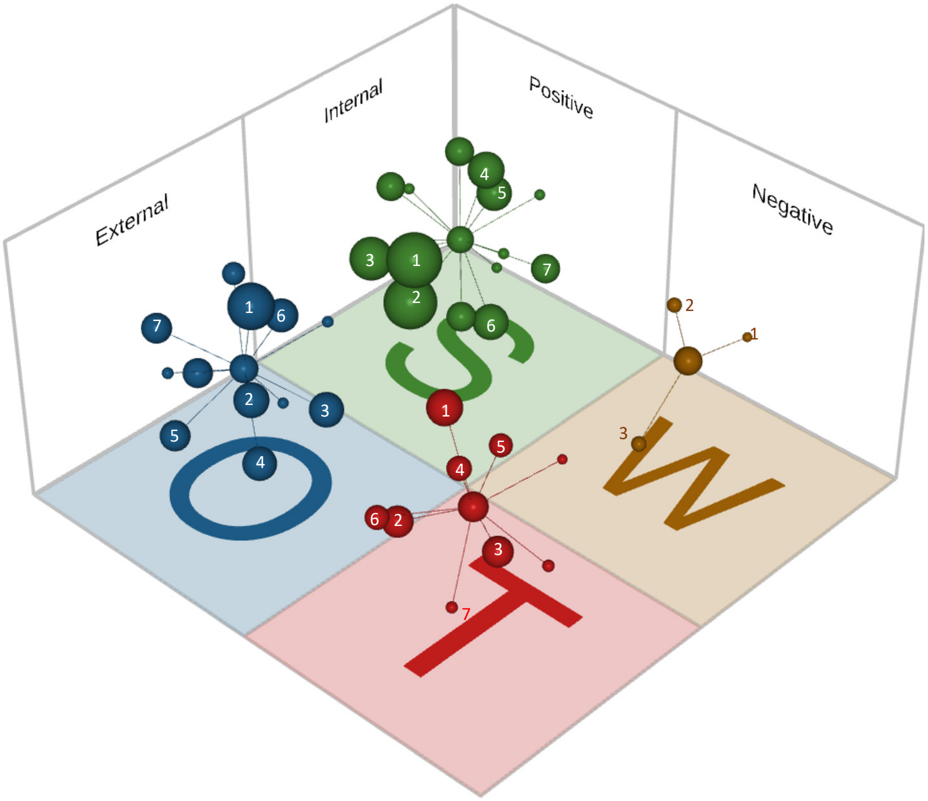

Figure A1. SWOT Bubble diagram.

No.	Strengths	Significance	Internal rating	Score	Weaknesses	Importance	Internal rating	Score	Opportunities	Importance	Likelihood	Score	Threats	Importance	Likelihood	Score
1.	"Effective learning platform to allow interaction between countries'	5	3	15	"Not enough time for discussions."	-4	1	-4	"Reduced travel stress"	3	3	9	"Different time zones"	-3	3	9
2.	"People who could not normally be part of international meetings could attend -socially just!"	5	3	15	"When we met in groups (compared individuals) there was more interaction before/after sessions"	-4	1	-4	"Reduced travel time"	3	3	9	"People could be distracted by household responsibilities, e.g. kids.	-4	2	8
3.	"Supporting and valuing Early Career Researchers."	4	3	12	"Sound was not always clear"	-1	1	-1	"Ability for people with home caring duties to partially join"	3	3	9	"Poor network (internet) issues could hamper participation by everyone."	-4	2	8
4.	"All countries could share views and knowledge and information in one 'room' without travel"	5	2	10					"Ability to still meet and keep work going despite the COVID-19 lockdown."	3	3	9	"In South Africa, electricity supply was interrupted (load shedding) leading to diconnection"	-3	2	6
5.	"Reduced carbon emissions"	5	2	10					"More younger people could participate"	3	3	9	"Lack personal interaction means we don't build collaborations"	-3	2	6
6.	"Reduces impact on environment" and "environmentally friendly"	5	2	10					"Chance to explore new ways of interacting with one another."	4	2	8	"Limitations for people with limited internet access"	-3	2	6
7.	"Cost effective and cheaper"	4	2	8					"Save my personal energy"	4	2	8	"Less sharing of individual ideas"	-3	1	3
8.	"Cost saving in terms of transport and catering" "savings used for future research"	4	2	8					"We should practice what we preach"	4	2	8	"Limited time meant new partnerships could not be formed"	-3	1	3
9.	"Could understand voices better because everyone had microphone"	4	2	8					"Annual meeting can be left online and be accessible in future""	3	2	6	"More difficult to interact electronically"	-2	1	2
10.	"People who cannot travel could join"	4	2	8					"Learn new technical knowledge"	3	1	3				
11.	"Equal participation from all three countries."	3	1	3					"We are showing global leadership on how to run virtual meetings" "the reality of future"	3	1	3				
12.	"Individual screens improve focus on presentations"	3	1	3					Everyone connecting in the same way means participation is equal"	3	1	3				
13.	"More people get to participate than just a few members from each country."	3	1	3												
14.	"More time to work on other things, less disruptive"	3	1	3												

Figure A2. SWOT Analyses Scores Table.

Appendix C

Table A1. List of actual attendees for the 2019 virtual meeting and proposed attendees for the face-to-face meeting. Attendees categorised into early-career/mid-career and senior level, male and female and Global South.

Location	Attendees Virtual Meeting		Proposed Attendees Face-to-Face Meeting	
	N	Level of Seniority	**N**	**Level of Senioritys**
**Pietermaritzburg**	27	15 senior researchers<br>12 early-career/mid-career researchers	10	9 senior researchers<br>1 early-career/mid-career researchers
**Durban**	26	4 senior researchers<br>21 early-career/mid-career researchers	5	5 senior researchers
**London**	23	8 senior researchers<br>15 early-career/mid-career researchers	19	8 senior researchers<br>11 early-career/mid-career researchers
**Aberdeen**	2	1 senior researchers<br>1 early careerng/mid-career researchers	2	1 senior researchers<br>1 early-career/mid-career researchers
**Delhi**	6	2 senior researchers<br>4 early-career/mid-career researchers	3	2 senior researchers<br>1 early-career/mid-career researchers
**Bangalore**	16	9 senior researchers<br>7 early-career/mid-career researchers	10	4 senior researchers<br>6 early-career/mid-career researchers
**Other**	7	0 senior researchers<br>7 early-career/mid-career researchers	0	--<br>--
**TOTAL**	107	47 senior researchers<br>67 early careerg/mid-career<br>68 female, 36 male<br>75 Global South (31 senior; 44 early-career/mid-career, 53 female)	49	28 senior<br>21 early-career/mid-career<br>29 female, 20 male<br>28 Global South (19 senior; 9 early-career/mid-career, 16 female)

Appendix D

Survey Themes and Sub-Themes

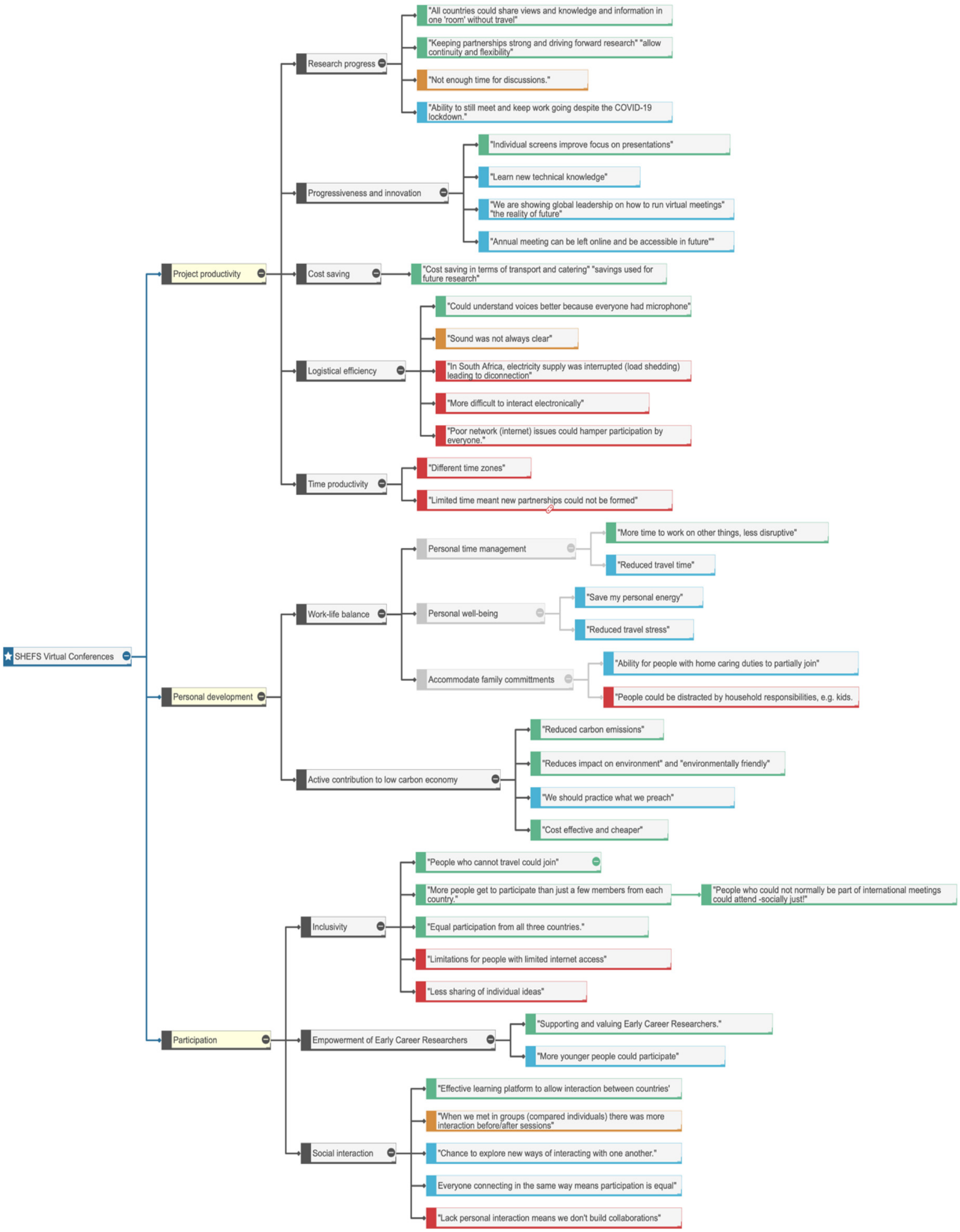

Figure A3. The figure shows the themes and sub-themes that were identified from survey responses in 2019 and 2020, before and after the two virtual conferences. Three main themes identified were: (1) Project productivity; (2) Personal Development and (3) Participation. For each sub-theme, comments were extracted for each category of the Strength (green boxes), Weakness (orange boxes), Opportunities (blue boxes) and Threats (red boxes) (SWOT).
